# 1-Methylcyclopropene Preserves the Quality of Chive (*Allium schoenoprasum* L.) by Enhancing Its Antioxidant Capacities and Organosulfur Profile during Storage

**DOI:** 10.3390/foods10081792

**Published:** 2021-08-02

**Authors:** Xiaomei Dai, Yaping Lu, Yuan Yang, Zhifang Yu

**Affiliations:** 1 College of Food Science and Technology, Nanjing Agricultural University, Nanjing 210095, China; 2019208001@njau.edu.cn (X.D.); 2020808125@stu.njau.edu.cn (Y.Y.); 2 Department of Food Science and Technology, Jiangsu Food & Pharmaceutical Science College, Huaian 223003, China; 3 College of Life Science, Nanjing Agricultural University, Nanjing 210095, China; lyphwq@njau.edu.cn

**Keywords:** chive, senescence, quality, antioxidant capacity, amino acids, organosulfur compounds

## Abstract

The quality, antioxidant capacities, and organosulfur profile of chives (*Allium schoenoprasum* L.) treated with 1-methylcyclopropene (1-MCP) during storage were investigated in this study. The 1-MCP treatment (100 μL/L, fumigation 12 h at 20 °C) effectively inhibited tissue respiration and H_2_O_2_ production, enhanced the ascorbic acid (ASA) and glutathione (GSH) content, and promoted the activity of antioxidant enzymes (superoxide dismutase SOD, Catalase CAT, and ascorbic peroxidase APX) during the 5-day storage period at 20 °C. The result further showed that the 1-MCP treatment inhibited chlorophyll degradation, alleviated cell membrane damage, and delayed the chive senescence, with the yellowing rate being reduced by 67.8% and 34.5% in the 1-MCP treated chives on days 4 and 5 of storage at 20 °C, respectively. The free amino acid content of the chive was not affected by the 1-MCP treatment at 20 °C. However, the senescence rate of the chive was not reduced by the 1-MCP treatment when stored at 3 °C. The liquid chromatography data further showed that the 1-MCP treatment induced a 15.3% and 13.9% increase in the isoalliin and total S-alk(en)ylcysteine sulfoxides (ACSOs) content of the chive on day 2 at 20 °C, respectively. Furthermore, there was a strong positive correlation between ACSOs content and CAT/APX activity, indicating that ACSOs probably played a key role in enhancing the antioxidant capacities of the chive during storage at 20 °C. Thus the study efficiently demonstrates that 1-methylcyclopropene preserves the quality of chive (*Allium schoenoprasum* L.) by enhancing its antioxidant capacities and organosulfur profile during storage.

## 1. Introduction

The *Allium* genus includes several hundred species and is widely utilized as a drug in folk medicine for its potent antibacterial, hypoglycemic, hypolipidemic, hypocholesterolemic, cardiovascular, antithrombotic, and antitumor activities [1,2,3,4]. *Allium schoenoprasum* L (chive) is rich in carbohydrates, proteins, amino acids, vitamins, and minerals and is cultivated as vegetables or seasoning herbs all around the world [5]. Like other *Allium* plants, chives have a unique flavor, and the precursor for flavor and therapeutic compounds are S-alk(en)ylcysteine sulfoxides (ACSOs) [6].

Similar to many green leafy vegetables, postharvest chives have a high respiration and senescence rate which often leads to quality deterioration, including leaf yellowing, wilting, and nutrient loss at ambient temperature storage. The most obvious characteristics of senescence are degradation of chlorophyll, breakdown of chloroplasts, increased oxidation, and hydrolysis of macromolecules, such as proteins [7]. During postharvest storage, leaf senescence is induced by different stress conditions, which culminate in an increased level of reactive oxygen species, ROS (including O_2_^−^, H_2_O_2_, and·OH^−^) [1] and its concomitant oxidative reaction. In addition to their destructive effect on cells, ROS can act as signaling molecules, by enhancing the antioxidant system for scavenging ROS [8]. When ROS homeostasis is broken down, membrane-lipid or protein oxidative damage occurs [9]. Research on the senescence mechanism of chive is very important for prolonging the shelf life of the *Allium* genus plant.

The current consensus is that the postharvest treatment-dependent delay in leaf senescence in the vegetables is mostly attributed to decreased ROS production [10]. To date, postharvest 1-MCP treatment has proven to be effective in extending the postharvest life of a wide range of vegetables, such as Chinese kale [11], broccoli [12], and pak choi [13]. The mechanism of 1-MCP delay in senescence of vegetables is not only by inhibiting ethylene action but also by enhancing stress response and defense [14], such as enhancing antioxidant enzyme activity [15]. The effect of 1-MCP treatment on the postharvest quality of the *Allium* genus has been rarely reported in the literature. Thus, a study on the effect of 1-MCP treatment on the storage quality of chive will be practically useful in the postharvest management of the *Allium* genus plant.

The physiological and biochemical properties of similar plant species such as garlic and onion have been extensively studied [16,17,18,19,20]. However, there are few literature reports on the relationship between S-alk(en)ylcysteine sulfoxides (ACSOs) and antioxidant capacity in the *Allium* plant during storage. The objective of this study is to determine the impact of 1-MCP treatment on leaf quality, antioxidant capacity, and organosulfur compounds of chive during storage at temperatures (20 °C and 3 °C). The relationship between ACSOs and the antioxidant capacity of the chive leaf was also studied. The results of this work will provide insight into the mechanisms of senescence physiology in *Allium* vegetables as well as the role of ACSOs in *Allium* vegetable senescence.

## 2. Materials and Methods

### 2.1. Materials and Treatments

The chive (*Allium schoenoprasum* L. cv. Xinghua), grown in Zhujia village, Xinghua County (nationally the most condensed cultivating area), Jiangsu Province, China, was used for this study. Harvested chives (day 0) were transported to the laboratory within 3 h at 20 °C. The senescent leaves were removed while the leaves with uniform sizes were selected and dried with a fan. The sample was then separated into four groups, CK 20 °C (non-treated), CK 3 °C (non-treated), 1-MCP 20 °C and 1-MCP 3 °C (treated with 1-MCP).

In the preliminary study, the chive leaves were fumigated with 1, 5, 10, 50, 100, 200 and 500 μL/L 1-MCP for 12 h at 20 °C and stored for 5 d at 20 °C with 85–90% humidity. The results indicated that 100 μL/L 1-MCP treatment was the most effective in delaying senescence in the leaf. Thus, 100 μL/L 1-MCP was used to fumigate the sample for 12 h at 20 °C. Every 4 kg chives were fumigated in a plastic container. A Petri dish containing 1% (*w*/*v*) KOH aqueous solution was kept inside the plastic container to prevent the accumulation of CO_2_ by chives. After fumigation (day 0.5), the CK 20 °C group and 1-MCP 20 °C group were stored at 20 °C (RT) with 85–90% humidity for 5 d, while the CK 3 °C group and 1-MCP 3 °C group were stored at 3 °C (LT) with 85–90% humidity for 20 d (Table 1).

At every testing point, 3 kg chives were sampled in each group in three biological replicates (1 kg chives per replicate) for quality parameter analysis, physiology and ROS level. After the above determinations, samples of size 0.5–1 cm were collected, immediately frozen in liquid nitrogen, and stored at −80 °C for further analysis. As the white shaft part was very short, chives were sampled starting from the white and green transition to 2 cm of the top of the leaves, which represent the whole plant.

### 2.2. Reference Compounds

S-methyl-L-cysteine sulfoxide (MCSO, CAS:6853-87-8, HPLC ≥ 98%) was purchased from Nanjing Chemlin Chemical Industrial Co., Ltd.; S-allyl-L-cysteine sulfoxide (ACSO, CAS:556-27-4, HPLC ≥ 98%) was purchased from Shanghai Yuanye Bio-Technology Co., Ltd.; S-propyl- L-cysteine sulfoxide (PCSO, CAS:17795-24-3, HPLC ≥ 98%) was purchased from Jiangsu Aikon Biopharmaceutical R&D Co., Ltd.; (E)-S-(1-propenyl)- L-cysteine sulfoxide (PeCSO) was identified by high-resolution mass spectrometry (MS). Twelve amino acids, asparagine (Asn), glutamine (Gln), serine (Ser), arginine (Arg), threonine (Thr), alanine (Ala), proline (Pro), methionine (Met), valine (Val), tryptophan (Trp), leucine (Leu), phenylalanine (Phe), HPLC ≥ 98%) were all purchased from Shanghai Yuanye Bio-Technology Co., Ltd.

### 2.3. Determination of Chlorophyll Content and Yellowing Rate

Chlorophyll content was measured as reported by [21] with slight modifications. Chive tissue (0.5 g) was ground with 5 mL of 95% ethanol (*v*/*v*) and then centrifuged at 12,000 g for 15 min at 4 °C. The chlorophyll content was quantified by measuring the absorbances at 665 and 649 nm in the supernatant and the content was expressed as g kg^−1^. Except for the content of free amino acids, PeCSO and ACSOs, all the other results were expressed on a fresh weight basis.

At each time point, 1 kg chives from each sampling treatment were used to measure the yellowing rate. The leaf yellowing scales were divided into five grades: grade 0: no yellowing area; grade 1: approximately 1–10% yellowing area; grade 2: approximately 10–25% yellowing area; grade 3: approximately 25–50% yellowing area; and grade 4: more than 50% yellowing area. Leaf yellowing rate = ∑ (yellowing grade × number of leaves at this grade)/(total number of leaves × the highest grade).

### 2.4. Determination of Respiration Rate, MDA and H_2_O_2_

Respiration rate was directly measured by a CO_2_ gas analyzer (CheckMate 3, Dansensor, Denmark). Approximately 400 g chives from each biological replicate were enclosed in 7.4 L glass jars at 20 °C or 3 °C for 1 h to measure respiration rate.

Hydrogen peroxide (H_2_O_2_) content and malondialdehyde (MDA) content were measured using the method of [22]. H_2_O_2_ content and MDA content were expressed as mmol kg^−1^.

### 2.5. Determination of Antioxidants

ASA concentration was determined by the method as described by [23] and was expressed as g kg^−1^. GSH (reduced glutathione) and GSSG (oxidized glutathione) concentrations were measured by an enzymatic cycling assay method described by [24] and were expressed as μmol kg^−1^.

### 2.6. Determination of Antioxidant Enzymes

Superoxide dismutase (SOD) and ascorbic peroxidase (APX) activity measurements were adapted from [25]. One unit of SOD activity was defined as the amount of enzyme that caused 50% inhibition of nitro blue tetrazolium (NBT) at 560 nm. One unit of APX enzyme activity was defined as a decrease in absorbance by 0.001 per minute at 290 nm under the assay conditions.

Catalase (CAT) activity was determined according to the method reported by [26] with a slight modification. Chive tissue (0.5 g) was ground using 6 mL phosphate buffer (0.1 mol/L, pH 7.5, containing 5 mmol/L 1,4-Dithiothreitol and 5% polyvinyl pyrrolidone). The reaction mixture consisted of 2.8 mL sodium phosphate buffer (50 mmol/L, pH 7.5, containing 20 mmol/L H_2_O_2_) and 0.2 mL enzyme extract, with a total volume of 3.0 mL. H_2_O_2_ decomposition was measured by the decrease in absorbance at 240 nm. One unit was defined as the amount of enzyme that caused one absorbance change per minute under the assay conditions.

Peroxidase (POD) activity was assayed as previously described by [27]. POD activity was measured based on guaiacol oxidation by H_2_O_2_ at 470 nm. The absorbance at 470 nm was recorded every 30 s using a spectrophotometer. One unit of enzyme activity was defined as the amount of enzyme required to increase the absorbance by 0.001 min^−1^ under the assay conditions.

### 2.7. Determination of GTP and Allinase

The γ-glutamyl transpeptidase (GTP) activity was determined according to the method reported by [28] with a slight modification. The 1 g samples were ground with 5 mL pre-cooled Tris-HCl (0.05 mol/L, containing 1 mol/L NaCl, 5 mmol/L 6-aminocaproic acids, 1 mmol/L phenylmethanesulfonyl fluoride) buffer at 4 °C, centrifuged at 12,000 g for 15 min at 4 °C. The reaction mixture, containing 0.2 mL enzyme extracts, 1 mL of 4 mmol/L γ-glutamyl p-nitroanilide and 1 mL of 40 mmol/L L-methionine, was incubated at 37 °C for 30 min. The absorbance was measured at 410 nm against a blank (enzyme extract substituted by distilled water). One unit of GTP activity is defined as the amount of enzyme that liberates 1 μmol of nitroaniline per min under standard conditions.

Allinase activity was determined according to the method reported by [29] with a slight modification. The 1 g samples were ground with 5 mL pre-cooled (4 °C) phosphate buffer (50 mmol/L, containing 5 mmol/L EDTA-Na_2_, 25 μmol/L pyridoxal phosphate, pH 7.0), then centrifuged at 12,000 g for 15 min at 4 °C. The reaction mixture was 0.1 mL above enzyme extracts and 10 mmol/L S-methyl cysteine sulfoxide. The reaction mixture was incubated at 37 °C for 10 min. The enzymatic reaction was terminated by the addition of 1 mL of 2 mol/L HCl containing 5 mmol/L dinitrophenyl hydrazine. Subsequently, the mixture was incubated at 37 °C for 5 min, afterwards, 5 mL of 2.5 mol/L NaOH was added to the solution, incubated for 10 min and the absorbance was measured at 420 nm against a blank (enzyme extract substituted by distilled water). One unit of allinase activity is defined as the amount of enzyme that produces 1 μmol pyruvate per min under standard conditions.

### 2.8. Determination of Organosulfur Compounds and Amino Acids

The organosulfur compounds and amino acids were extracted and determined according to the method reported by [30] with a slight modification. An amount of 10 g chives were steamed with boiling water for 8 min, after being cooled, ground into a powder with a blender in liquid nitrogen and carefully transferred to a glass container. The sample powder was extracted with 100 mL 90% aqueous methanol containing 10 mmol/L HCl for 20 min with a magnetic stirrer at 40 °C, filtered and the residue was extracted with another 100 mL methanol again for 10 min as the same operation above. The combined methanolic extracts were concentrated at reduced pressure (at 40 °C) to approximately 10–15 mL and adjusted to 25 mL by 20 mmol/L borate buffer (pH 9.2). This extract was stored in the refrigerator (−20 °C) until derivatization.

Dansyl derivatives were prepared by mixing 100 μL of the sample extract with 250 μL of the dansyl chloride (Dns-Cl) reagent (10 mmol/L Dns-Cl in acetonitrile) and 0.65 mL of 20 mmol/L borate buffer (pH 9.2). The mixture was briefly shaken, allowed to stand at room temperature for 30 min, filtered through a 0.45 μm nylon filter, and analyzed by HPLC.

A high-performance liquid chromatography (HPLC) (LC-20AD, Shimadzu, Japan) equipped with a ZORBAX SB-Aq (Agilent, USA, 4.6 × 250 mm, 5 μm) column and PDA detector (SPD-M20A, Shimadzu, Japan) was used to determine the content of organosulfur compounds and amino acids. The chromatographic condition was as follows: 50 mmol/L sodium acetate buffer (pH 5.0, solvent A) and methanol (solvent B) were used as the mobile phase, with a flow rate of 0.9 mL min−1 and the gradient A/B 70/30 (0 min), 70/30 (2 min), 60/40 (in 12 min), 50/50 (in 32 min), 40/60 (in 42 min), 5/95 (in 42.01 min), 5/95 (in 51 min), 70/30 (in 51.01 min), and 70/30 (in 60 min), detection wavelength of 250 nm. The injection volume was 20 μL. The content of free amino acids, PeCSO and ACSOs, were expressed on a dry weight basis.

For identification of each peak, the eluents corresponding to each peak of HPLC were collected separately, desalted by C-18 solid-phase extraction column and analyzed by LC-MS system (G2-XS QTof, Waters). A 2 μL solution was injected into the UPLC column (2.1 × 100 mm ACQUITY UPLC BEH C18 column containing 1.7 μm particles) with a flow rate of 0.3 mL/min. Buffer A consisted of 0.1% formic acid in the water, and buffer B consisted of 0.1% formic acid in acetonitrile. The gradient was 5% buffer B for 0.5 min, 5–95% buffer B for 11 min, 95% buffer B for 2 min.

Mass spectrometry was performed using an electrospray source in positive ion mode with MS acquisition mode, with a selected mass range of 50–1200 m/z. The lock mass option was enabled using leucine-enkephalin (m/z 556.2771) for recalibration. The ionization parameters were the following: the capillary voltage was 2.5 kV, sample cone was 40 V, source temperature was 120 °C (and desolvation gas temperature was 400 °C). Data acquisition and processing were performed using Masslynx 4.1.

### 2.9. Statistical Analysis

All values are reported as the mean ± standard errors of three biological replicates. Statistical analysis was performed with the SPSS 18.0 software (SPSS Inc, Chicago, IL, USA). The significant differences between mean values were analyzed using the LSD’s test (*p* < 0.05). Origin 2021 (Microcal Software, Northampton, MA, USA) was employed to create figures.

## 3. Results

### 3.1. Yellowing Rate, Chlorophyll Content and Appearance Analysis

**Figure 1:**
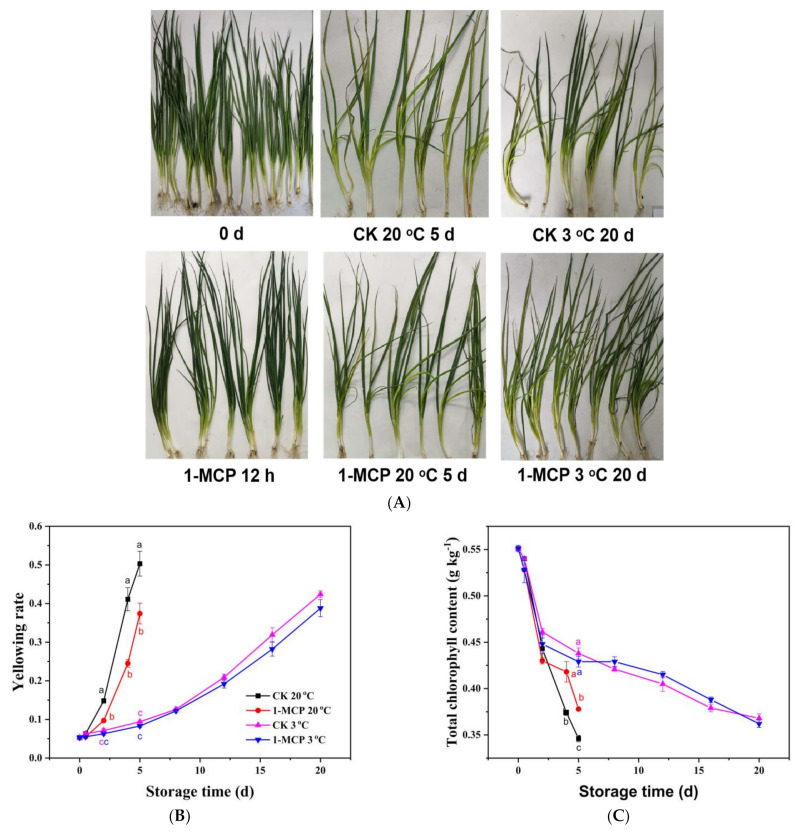
Appearance (**A**), yellowing rate (**B**) and total chlorophyll content (**C**) of 1-MCP treated and untreated chives during storage at 20 °C and 3 °C. Data are expressed as the mean of triplicate samples. Vertical bars represent the standard errors of the means. The different letters at each time point indicate a difference among the chives samples.

The yellowing rate is a sensory quality while chlorophyll content is a physiological index used to assess the color status of chives. As shown in Figure 1, chives gradually lost the green color and turned yellow during storage. Leaves in the CK group were more yellow than 1-MCP treated chives on day 4 and day 5 during storage at 20 °C. In agreement with the appearance, the content of chlorophyll in all groups decreased constantly during storage, and the yellowing rate increased progressively. The chlorophyll content was 11.8% and 9.2% higher in 1-MCP treated samples on day 4 and day 5, respectively, than that in the CK group during RT storage. The yellowing rate of 1-MCP treated chives was 67.8% and 34.5% lower than that of CK samples on day 4 and day 5, respectively, during RT storage and negatively correlated with the chlorophyll content (r = −0.89 **). However, there was no difference in both the yellowing rate and the chlorophyll content of the 1-MCP treated and CK chives during storage at 3 °C. The result suggests that 1-MCP treatment could delay degradation of chlorophyll in chives, maintain green color and prolong the shelf life during RT storage. Nonetheless, the 1-MCP treatment did not improve the storage quality of the chives during LT storage.

### 3.2. Respiration, H_2_O_2_ and MDA Analysis

**Figure 2:**
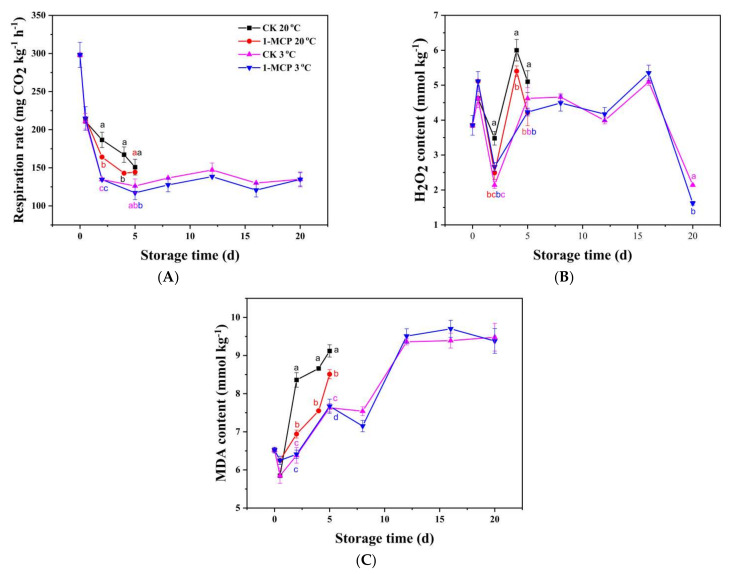
Respiration rate (**A**), H_2_O_2_ content (**B**) and MDA content (**C**) of 1-MCP treated and untreated chives during storage at 20 °C and 3 °C. Data are expressed as the mean of triplicate samples. Vertical bars represent the standard errors of the means. The different letters at each time point indicate a difference among the chives samples.

The respiration rate (Figure 2A) decreased sharply during the first 12 h of storage and then decreased at a slower rate afterward in all the experimental groups. Nevertheless, when stored at 20 °C, the respiration rate of 1-MCP treated chives was 13.7% and 17.0% lower than that of the CK group on day 2 and day 4. There was no difference between the respiration rate of the 1-MCP treated chives and the CK group stored during LT storage.

The H_2_O_2_ content in chives fluctuated, increasing at 12 h after harvest, dropping on day 2, and increased rapidly thereafter (Figure 2B). When stored at 20 °C, the H_2_O_2_ content in 1-MCP treated chives was 40.0% and 21.1% lower than that in the CK group on day 2 and day 5, respectively. However, when stored at 3 °C, the H_2_O_2_ content in the 1-MCP treated samples was higher than that in the CK group on day 2, with no difference afterward. This result suggested that 1-MCP treatment may have induced oxidative stress on chives transiently during LT storage.

The MDA contents, which represent an index used to assess the extent of oxidative damage to the membrane, increased progressively in all the groups during storage (Figure 2C). Nonetheless, the 1-MCP treatment reduced the MDA content in the chives compared to the CK during RT storage. The MDA content was 20.5% and 14.7% lower in the 1-MCP sample than that in the CK group on days 2 and 4 of storage at RT, respectively. The MDA content in samples stored in 3 °C increased sharply after 8 days, reaching a maximum on day 12, and did not change after that.

The results clearly show that the 1-MCP treatment suppressed respiration rate, inhibited H_2_O_2_ and MDA accumulation, and protected the membrane from oxidative damage during RT storage, while the 1-MCP treatment had no protective effect on the quality characteristics of the chives during LT storage.

### 3.3. Non-Enzymatic Antioxidants Analysis

**Figure 3:**
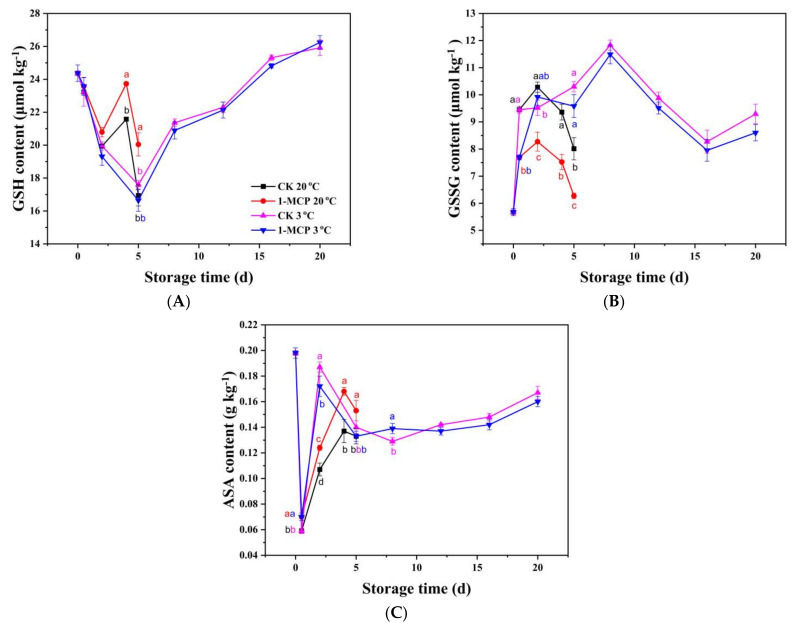
GSH content (**A**), GSSG content (**B**) and ASA content (**C**) of 1-MCP treated and untreated chives during storage at 20 °C and 3 °C. Data are expressed as the mean of triplicate samples. Vertical bars represent the standard errors of the means. The different letters at each time point indicate a difference among the chive samples.

As shown in Figure 3A,B, GSH content in chives presented an overall decreasing trend except increasing on day 4 during RT storage, while GSH content declined during the first 5 days, and then increased progressively during storage at LT. GSSG content increased firstly and decreased afterward during storage at both temperatures. The 1-MCP increased GSH content by 10.0% and 18.3%, respectively, on day 4 and day 5, and suppressed GSSG content from day 0.5 to day 5, with 24.4% and 27.8% lower rates in 1-MCP treated chives on day 4 and day 5.

With a negative correlation with GSSG (r = −0.71 **), ASA content (Figure 3C) in chives decreased sharply at the 12th hour, increased afterward and decreased again during storage. This might be due to the antioxidant activity of the ASA which culminated in reduce ROS accumulation, while the continuous synthesis of ASA might have been the reason for the fluctuation of ASA content. The 1-MCP treated chives had a higher ASA content during RT storage, 23.2% and 13.7% higher on day 4 and day 5 than untreated chives, respectively, while during LT storage, the 1-MCP treatment exerted a small effect on the ASA and GSH content of the chives.

### 3.4. Antioxidant Enzymes Analysis

**Figure 4:**
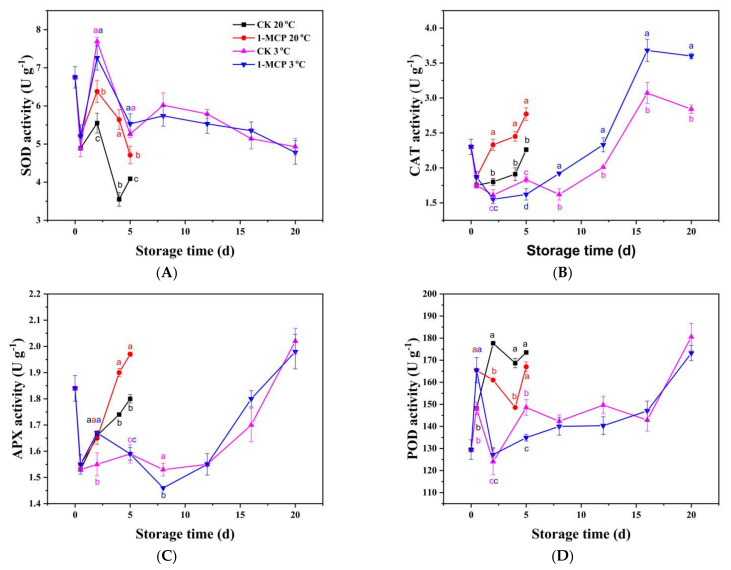
SOD activity (**A**), CAT activity (**B**), APX activity (**C**) and POD activity (**D**) of 1-MCP treated and untreated chives during storage at 20 °C and 3 °C. Data are expressed as the mean of triplicate samples. Vertical bars represent the standard errors of the means. The different letters at each time point indicate a difference among the chives samples.

Antioxidant enzymes in fruit and vegetables include SOD, CAT, APX, and POD. SOD is one of the most important antioxidant enzymes, and its activity declined in the first 12 h, increased afterward, dropped again during storage at RT. Nevertheless, SOD activity (Figure 4A) was 15.0%, 58.9% higher on day 2 and day 4, respectively, during RT storage in the 1-MCP treated sample than the CK. This result suggested that 1-MCP treatment could enhance SOD activity. The CAT activity (Figure 4B) and APX activity (Figure 4C) declined in the first 12 h after harvest in all groups, followed by a progressive increase. The CAT and APX showed a strong positive correlation (r = 0.84 **). When stored at 3 °C, the CAT and APX activity was unchanged from day 2 to day 8/12, after that it increased sharply at the late stage of storage in chives. The 1-MCP treatment improved the CAT activity in the treated sample during RT and LT storage. The 1-MCP treated chives had 29.4% and 28.3% higher CAT activity than untreated chives on day 2 and day 4, respectively, during RT storage, and 19.9% and 26.8% higher CAT activity than untreated chives on day 16 and day 20 during LT storage. The 1-MCP treatment also increased the APX activity during RT storage, with 9.2% and 9.4% higher APX activity in 1-MCP treated chives on day 4 and day 5. However, when stored at 3 °C, the APX activity in 1-MCP treated chives was higher than that in the CK group only on day 2; after that there was no difference in the APX activity. The results thus demonstrate that the 1-MCP treatment increased the activities of antioxidant enzymes in the chives during RT storage, but did not improve the antioxidant enzyme activities during LT storage.

There was no correlation between SOD/CAT/APX activity and POD activity. The POD activity increased dramatically in the first 12 h after harvest in all groups (Figure 4D). When stored at 20 °C, 1-MCP treatment inhibited the POD activity from day 2. When stored at 3 °C, the POD activity dropped on day 2, then increased from day 2 to day 16, with a more dramatic increase on day 20 for both the 1-MCP treated chives and the CK. The POD activity in the 1-MCP treated chives was higher on day 5 than that in CK, with no difference after that.

The results strongly demonstrate that 1-MCP treatment can efficiently enhance non-enzymatic and enzymatic antioxidant capacities in chives during RT storage, but do not affect the non-enzymatic and enzymatic antioxidant capacities of chives during LT storage.

### 3.5. Free Amino Acids Analysis

**Figure 5:**
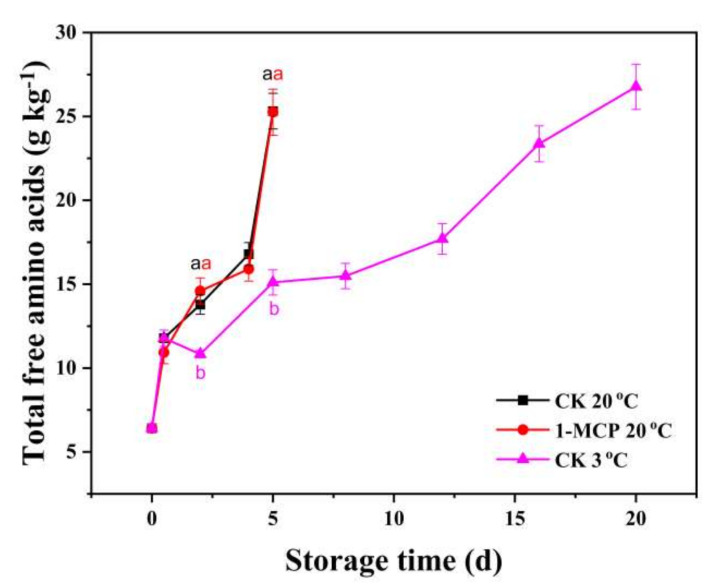
The content of total free amino acids of 1-MCP treated and untreated chives during storage at 20 °C and 3 °C. The content of free amino acids in 1-MCP treated chives stored at 3 °C was not determined. Data are expressed as the mean of triplicate samples. Vertical bars represent the standard errors of the means. The different letters of each treatment indicate a difference among the chives samples.

Based on the results of the preliminary experiment (1-MCP treatment had no effect on chives during LT storage), the content of ACSOs and free amino acids of 1-MCP treated chives stored at 3 °C were not determined. ACSOs and free amino acid content of the extract from chive samples are as shown in the HPLC chromatogram in Figure S1A. In postharvest vegetables, the degradation of protein, which is the result of vegetable senescence, will lead to an increase in the concentration of free amino acids. Generally, the concentration of almost all the identified free amino acids including Asn, Gln, Ser, Arg, Thr, Ala, Val, Trp, Leu, Phe in chive samples during storage at 20 °C and 3 °C was increased progressively, except Pro and Met (Figure S2). The content of total free amino acids increased at the 12th hour after harvest and further increased more dramatically at the end of storage, with a higher increase in their concentrations during RT storage, compared with LT storage (Figure 5). The Pro content in chives increased continuously when stored at RT but was not changed during storage at LT. A similar trend was observed in the Met content of the chives stored at 20 °C and 3 °C. Surprisingly, the 1-MCP treatment did not decrease the concentrations of total free amino acids during RT storage. The results thus show that the 1-MCP treatment could not inhibit the free amino acid content increasing.

### 3.6. Organosulfur Compounds and Related Enzymes Analysis

**Figure 6:**
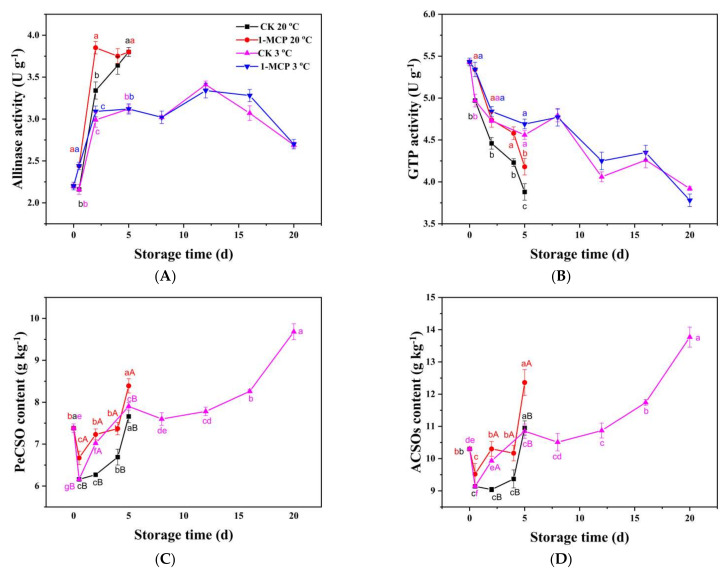
Allinase activity (**A**), GTP activity (**B**), PeCSO content (**C**) and ACSOs content (**D**) of 1-MCP treated and untreated chives during storage at 20 °C and 3 °C. The content of ACSOs in 1-MCP treated chives stored at 3 °C was not determined. Data are expressed as the mean of triplicate samples. Vertical bars represent the standard errors of the means. The lower and upper case letters indicate differences among the storage time and the treatments of the chive samples, respectively.

Allinase, which is located in the cell vacuoles, hydrolyzes ACSOs, producing a variety of antibacterial sulfur-containing compounds [6]. The allinase activity increased dramatically in the first two days after harvest in all groups, then changed less dramatically after that (Figure 6A). When stored at 20 °C, the allinase activity in 1-MCP treated chives was 13.0% and 15.3% higher than that in untreated chives after 12 h and on day 2, after that, there was no difference in allinase activity. The allinase activity in samples stored at 3 °C remained unchanged from day 2 to day 8, then increased on day 12 and finally decreased to the end of the storage. In contrast to allinase activity (r = −0.84 **), the GTP activity decreased progressively during storage. The GTP activity in the sample stored at 20 °C was observed to decrease more rapidly than that in the samples stored at 3 °C (Figure 6B). Nonetheless, the GTP activity was higher in 1-MCP treated samples than that in untreated samples during RT storage; while the 1-MCP treatment did not increase the allinase activity and maintain GTP activity in chives during LT storage.

Isoalliin (PeCSO) was identified using LC-MS coupled with high-resolution mass spectrometry, as shown in Figure S1B,C. S-alk(en)ylcysteine sulfoxides (ACSOs) are bioactive organosulfur compounds in chives and include alliin (ACSO), isoalliin (PeCSO), methiin (MCSO), propiin (PCSO) [6]. The PeCSO is the predominant component of ACSOs in chives, and change in the PeCSO content was observed to show a similar trend with ACSOs content during storage (r = 0.96 **) (Figure 6C,D). MCSO was the second major component of ACSOs in the chives (Table 2), with the ratio of 3:1 of PeCSO to MCSO, while the concentration of ACSO and PCSO were very low in the chives. The PeCSO and ACSOs content in all groups declined after 12 h but increased at the end of storage. The PeCSO and ACSOs content in 1-MCP treated samples was higher compared to the untreated chives during RT storage, with a 15.3% and 13.9% increase, respectively, on day 2. The PeCSO and ACSOs content in samples stored at 3 °C increase from the 12th hour to day 5 with no further increase until day 12. The PeCSO and ACSOs then surged higher after that, with a 26.4% and 25.8% increase at the end of the storage than that in the CK group stored at 20 °C.

## 4. Discussion

### 4.1. 1-MCP Enhances Antioxidant Capacity and Delays Senescence in Postharvest Chives

Through preliminary experiments, we found that chives could only be stored for 5 days at 20 °C and only 20 days at 3 °C. Thus we chose 5 days of study for 20 °C and 20 days of study for 3 °C. Vegetables do not require ethylene action for normal senescence to take place [31,32]. In this study, the ethylene concentration determined in chive was very low, with no significant difference between 1-MCP treatment and CK group under the condition by using Agilent GC system (6890N) and Agilent J&W GC columns (19095P-S23). Therefore, we did not show the result of ethylene production of chives.

Respiration is an indication of the tissue metabolic rate and the major factor that contributes to the deterioration and loss of quality of plant produce during postharvest storage [33]. Chive leaves, with a very high respiratory rate, suffer senescence very quickly after harvest. The 1-MCP treatment suppressed the respiration rate during RT storage, which is consistent with other reports [13,34]. Respiration, a basic biological process in a plant cell, produces reactive oxygen species (ROS) as an unavoidable by-product [8]. The 1-MCP treatment reduced the respiration rate and its accompanying ROS accumulation in the chives during storage at RT. This was evident in the lower H_2_O_2_ content in 1-MCP treated chives during RT storage. However, during LT storage, 1-MCP treated chives showed no difference in respiration rate and H_2_O_2_ content compared with untreated chives. This result strongly indicates that 1-MCP treatment does not affect the physical activity and enzyme activity of chive when stored at LT. Similar results have been reported in a combined 1-MCP and heat treatment on peach [34].

The MDA concentration and ROS level are often used as an index to measure membrane integrity and oxidative stress in vegetables [34]. In this study, the MDA concentration in 1-MCP treated samples was lower than that in untreated samples during RT storage. It is also found that the lower concentrations of H_2_O_2_ in 1-MCP treated vegetables correlated with the lower MDA level during RT storage. This finding suggests that 1-MCP treatment effectively maintained the membrane integrity of the chive by suppressing oxidative stress during RT storage, while the 1-MCP treatment did not affect the MDA and ROS levels of the chives during LT storage in this study. It has been reported that combined 1-MCP and heat-treatment even induced oxidative stress during LT storage in peach [34].

The 1-MCP preserved a high content of antioxidants (ASA and GSH) and high activities of antioxidant enzymes (SOD, CAT, and APX) in chives stored at 20 °C which might have contributed to scavenging ROS (H_2_O_2_), and maintained the cell structural integrity. The accumulation of ROS in plants during senescing can activate the enzymatic and non-enzymatic antioxidant system to scavenge ROS down to a safe range [35]. These results showed that 1-MCP treatment enhanced the antioxidant system during RT storage, thus, reduced ROS production. This postulation was validated by the fact that the H_2_O_2_ content and MDA content was lower in 1-MCP treated chives during RT storage. The 1-MCP treatment had little effect on the antioxidant system during LT storage, except for CAT activity. It has been reported in the literature that POD activity increases as the leaves are senescing, the role of which is dual [36]. On one hand, as a member of the protective enzyme system, it can clear reactive oxygen species; while on the other hand it participates in the degradation of chlorophyll and the production of reactive oxygen species, and triggers membrane lipid peroxidation, manifested as a damaging effect. In this study, 1-MCP treatment-induced POD activity in the first 12 h and inhibited it afterward during RT storage. Similar reports have found that hyperbaric pressures between 400 kPa and 800 kPa induced a reduction in POD activity due to the reduction in oxidative stress and delayed senescence in tomatoes [37]. Vacuum packing at pressures of down arrow 0.04 mPa at 4 °C has also been reported to suppress POD activity and maintained the quality of mung bean [38].

Yellowing, the main characteristic of leaf senescence, with a strong positive correlation with MDA (r = 0.89 **), is the result of chlorophyll degradation. The 1-MCP treatment delayed chlorophyll degradation in chives during RT storage. Similar findings have been reported [15,39]. The protective effects of 1-MCP on chlorophyll degradation may be attributed to the higher antioxidant ability and lower ROS level, maintaining membrane integrity [39]. Higher chlorophyll content in the 1-MCP treated chives correlated with lower H_2_O_2_ content on days 4 and 5 than that in the CK group during RT storage. The 1-MCP treatment on the chives during LT storage did not prevent chlorophyll degradation in this study. This might be due to the inability of the 1-MCP treatment to decrease oxidative stress and maintain membrane integrity of the chive during LT storage.

When plant cells undergo senescence, they suffer from oxidative stress and produce a large amount of ROS, causing membrane lipid peroxidation, degradation of protein, and production of free amino acids [40,41]. The total free amino acids increased progressively at first, then increased more dramatically the last day (RT storage) and on the last 8 days during LT storage (Figure 5), which was an indication that the chloroplast had been severely degraded. This was also evident in the strong positive correlation between the content of free amino acids and MDA (r > 0.7 **), the content of free amino acids and yellowing rate (r > 0.8 **), and the strong negative correlation between free amino acids and chlorophyll content (r > −0.8 **). A similar result has been reported on the increase in free amino acids in mushrooms during postharvest storage [42]. In this study, the 1-MCP treatment did not affect the accumulation of free amino acids in the chives during storage which is similar to the report that both ice and the novel phase change material treatment to *Pleurotus eryngii* showed no effects on total free amino acids [43].

In summary, the results indicate that 1-MCP enhances antioxidant capacity, reduces oxidative stress, and delays senescence in postharvest chives during storage at RT, while 1-MCP treatment had no effect on the antioxidant capacity, oxidative stress, and senescence levels on chives during storage at LT.

### 4.2. ACSOs Probably Act as Antioxidants Being Increased by 1-MCP in Postharvest Chives

S-alk(en)ylcysteine sulfoxides (ACSOs) are bioactive compounds that are beneficial for both the plant itself and human health due to their antibiotic properties. It has been widely reported that alliin is an effective hydroxyl (OH) scavenger [44,45,46], it efficaciously scavenged ·OH more than ascorbic acid [45], and scavenged superoxide generated by the xanthine/xanthine oxidase system [44]. It has been reported that the content of pyruvic acid, the by-product of ACSOs enzymatically hydrolyzed by allinase, was strongly correlated with increased FRAP (ferric reducing antioxidant power) activity [47]. Some researchers have also reported garlic had the highest content of total organosulfur compounds (mainly alliin) during the 8 weeks of storage which positively correlated with the maximum antioxidant capacities exhibited at 8 weeks of the storage [48]. In this study, the content of PeCSO and ACSOs showed a strong positive correlation with CAT activity (r = 0.86 **, 0.80 **) and with APX activity (r = 76 **, 0.72 **), respectively, indicating that ACSOs in chives probably play a key role in antioxidant capacities during storage. ACSOs in chives increased during storage, and 1-MCP treatment induced an increase in the ACSOs content during RT storage. LT storage induced an increase in ACSOs, with higher ACSOs in chives stored at 3 °C than that in 20 °C at the end of storage. Similar studies have also observed an increase in the total amount of the major ACSOs (e.g., alliin, methiin, and isoalliin) of up to 30% when garlic bulbs of 58 genotypes were stored at 5 °C [49]. It has been found that after 5 months of cold storage, ACSOs and enzymatically produced pyruvic acid concentrations increased in 9 of 10 genotypes of onion [20]. Some reports [50] have suggested that the increase in pungency during storage is due to the release of (1-propenyl)-L-cysteine sulfoxide (isoalliin) from γ-glutamylcysteine sulfoxide by γ-glutamyl transpeptidase (GTP), which increases its activity up to five-fold just before the onion starts sprouting, while in this study, GTP activity declined progressively during storage in chives. The increase in ACSOs content with a decrease in GTP activity suggests that perhaps enzymes possibly exist which could hydrolyze γ-glutamylcysteine sulfoxide to produce ACSOs. It also further suggests that the ACSOs could have been biosynthesized from other compounds such as amino acids. [51]. There are two proposed routes for alliin biosynthesis, one is from glutathione via γ-glutamyl peptides, while the other is from serine and allyl thiol [6]. In this study, two sulfur amino acids, cysteine of a very low concentration, and methionine, which its concentration decreased during storage while increasing at the end of storage, may be involved in the biosynthesis of ACSOs. This further suggests that the GTP might not have been involved in the biosynthesis of ACSOs in the chive samples. Allinase is a protective enzyme that hydrolyzes ACSOs to produce bioactive compounds when the tissue is attacked by insects or a pathogen. Its activity increased sharply from day 2 of the storage and maintained high activity throughout the storage period under RT. However its activity declined on day 20 during storage at low temperature. This result suggested that allinase activity decreased at the end of LT storage as its physical activity declined. The normalized transcription of allinase enzymes has been reported to be very high during sprouting but declined at the senescent stage of leaves when the alliin had transferred to the bulb [52]. This indicates that the allinase activity can be induced by biotic or abiotic stress for self-protection and survival in plants. The 1-MCP treatment induced higher allinase activity in chives during RT storage, while it had little effect on chives’ allinase activity during LT storage.

Overall, ACSOs showed a strong positive correlation with the CAT and APX, indicating that ACSOs may act as antioxidants in chives. The 1-MCP increased the content of ACSOs during storage at RT. However, compared with RT storage, chives stored at LT storage had higher ACSOs content.

## 5. Conclusions

**Figure 7:**
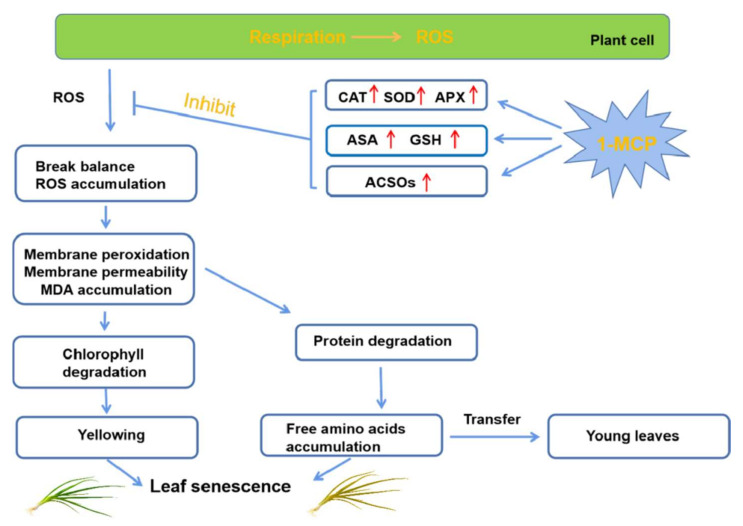
Possible senescence mechanism of chive leaves and possible mechanism of yellowing inhibition of chives during storage by 1-MCP treatment.

The possible senescence mechanism of chive leaves and mechanism of yellowing inhibition of chives during storage by 1-MCP treatment is shown in Figure 7. The 100 μL/L 1-MCP treatment was effective in inhibiting ROS production, enhancing antioxidant ability, reducing membrane damage, delaying chive yellowing and senescence during RT storage. Compared with RT storage, 1-MCP treatment had no positive effect on delaying chive senescence during LT storage. However, the 1-MCP treatment (under RT) and LT storage (compared with RT storage) promoted the increase of ACSOs content in the chives during storage. The present study indicates that ACSOs in chives probably play a key role in antioxidant activity due to their strong positive correlation with the increase in antioxidant enzyme activities during storage. To the best of our knowledge, this is the first report on the correlation between ACSOs and antioxidant ability. In conclusion, 1-MCP treatment is more effective in improving chives quality and delaying senescence for chives stored at RT, compared to chives stored at LT. Thus the study efficiently demonstrates that 1-methylcyclopropene preserves the quality of chive (*Allium schoenoprasum* L.) by enhancing its antioxidant capacities and organosulfur profile during storage.

## Figures and Tables

**Table 1:** Treatments and storage conditions of chive vegetables in four groups.

Group	Treatment	Storage Condition	Sample Time
CK 20 °C	No	20 °C	Day 0, 0.5, 2, 4 and 5
1-MCP 20 °C	1-MCP	20 °C	Day 0, 0.5, 2, 4 and 5
CK 3 °C	No	3 °C	Day 0, 0.5, 2, 5, 8, 12, 16 and 20
1-MCP 3 °C	1-MCP	3 °C	Day 0, 0.5, 2, 5, 8, 12, 16 and 20

Note: Day 0 means the day on harvest; Day 0.5 means at the time when 1-MCP fumigation for 12 h was just completed.

**Table 2:** S-alk(en)ylcysteine sulfoxides (ACSOs) content in chives.

Compounds Name	Storage Period (d)	0	0.5	2	4	5	8	12	16	20
MCSO (g kg^−1^)	CK 20 °C	2.44 ± 0.08 a	2.23 ± 0.00 b	2.10 ± 0.02 c,C	1.99 ± 0.09 d	2.16 ± 0.07 b,B	nd	nd	nd	nd
	1-MCP 20 °C	2.44 ± 0.08 b	2.12 ± 0.10 c	2.40 ± 0.08 b,A	2.08 ± 0.08 c	2.70 ± 0.12 a,A	nd	nd	nd	nd
	CK 3 °C	2.44 ± 0.08 b	2.23 ± 0.00 c,d	2.25 ± 0.01 c,B	nd	2.12 ± 0.14 d,e,B	2.04 ± 0.10 e	2.15 ± 0.13 c,d,e	2.52 ± 0.02 b	2.85 ± 0.11 a
ACSO (g kg^−1^)	CK 20 °C	0.35 ± 0.01 c	0.62 ± 0.06 b,A	0.57 ± 0.04 b	0.57 ± 0.06 b	1.01 ± 0.04 a,A	nd	nd	nd	nd
	1-MCP 20 °C	0.35 ± 0.01 c	0.41 ± 0.04 c,B	0.56 ± 0.03 b	0.62 ± 0.05 b	1.06 ± 0.09 a,A	nd	nd	nd	nd
	CK 3 °C	0.35 ± 0.01 e	0.62 ± 0.06 d,A	0.56 ± 0.03 d	nd	0.72 ± 0.05 c,B	0.75 ± 0.02 c	0.82 ± 0.06 b	0.83 ± 0.07 b	1.07 ± 0.09 a
PCSO (g kg^−1^)	CK 20 °C	0.13 ± 0.00 a	0.12 ± 0.00 b,B	0.11 ± 0.00 d,B	0.11 ± 0.01 d,A	0.12 ± 0.00 c	nd	nd	nd	nd
	1-MCP 20 °C	0.13 ± 0.00 b	0.23 ± 0.01 a,A	0.12 ± 0.01 c,A	0.09 ± 0.01 d,B	0.12 ± 0.01 b,c	nd	nd	nd	nd
	CK 3 °C	0.13 ± 0.00 b	0.12 ± 0.00 c,B	0.10 ± 0.00 d,B	nd	0.12 ± 0.00 c	0.09 ± 0.01 e	0.12 ± 0.00 c	0.13 ± 0.00 b	0.16 ± 0.01 a
PeCSO (g kg^−1^)	CK 20 °C	7.38 ± 0.17 a	6.16 ± 0.01 c,B	6.27 ± 0.06 c,B	6.69 ± 0.32 b,B	7.66 ± 0.26 a,B	nd	nd	nd	nd
	1-MCP 20 °C	7.38 ± 0.17 b	6.67 ± 0.27 c,A	7.23 ± 0.22 b,A	7.38 ± 0.25 b,A	8.39 ± 0.29 a,A	nd	nd	nd	nd
	CK 3 °C	7.38 ± 0.17 e	6.16 ± 0.01 g,B	7.02 ± 0.04 f,A	nd	7.90 ± 0.16 c,B	7.60 ± 0.26 d,e	7.78 ± 0.17 c,d	8.26 ± 0.07 b	9.68 ± 0.33 a
Total ACSOs (g kg^−1^)	CK 20 °C	10.30 ± 0.03 b	9.14 ± 0.07 c	9.04 ± 0.12 c,B	9.37 ± 0.48 c,B	10.95 ± 0.37 a,B	nd	nd	nd	nd
	1-MCP 20 °C	10.30 ± 0.03 b	9.43 ± 0.58 c	10.30 ± 0.39 b,A	10.17 ± 0.42 b,A	12.27 ± 0.69 a,A	nd	nd	nd	nd
	CK 3 °C	10.30 ± 0.03 d,e	9.14 ± 0.07 f	9.93 ± 0.07 e,A	nd	10.85 ± 0.38 c,B	10.48 ± 0.47 c,d	10.87 ± 0.40 c	11.74 ± 0.16 b	13.77 ± 0.53 a

**Note:** nd means not detect. Total ACSOs content is the sum of the content of MCSO, ACSO, PeCSO, and PCSO. Data are expressed as the mean of triplicate samples ± standard errors. The lower and upper case letters indicate differences among the storage time and the treatments of the chive samples, respectively.

## Data Availability

Not applicable.

## Supplementary Materials

The following are available online at https://www.mdpi.com/article/10.3390/foods10081792/s1, Figure S1: Separation and identification of organosulfur compounds and amino acids. HPLC chromatograms of organosulfur compounds and amino acids in chives (A), the peaks as the following: 1.Asn, 2. Gln, 3. Ser, 4. MCSO, 5. Arg, 6. Thr, 7. Ala, 8. PeCSO, 9. ACSO, 10. PCSO, 11. Pro, 12. Met, 13. Val, 14. Trp, 15. Leu, 16. Phe. TIC (B) and Mass spectrum (C) ([M+H]^+^) of LC-MS chromatograms of separated peak 8 (a derivative of isoalliin with Dns-Cl) of chives extracts., Figure S2: Asn content (A), Gln content (B), Ser content (C), Arg content (D), Thr content (E), Ala content (F), Val content (G), Trp content (H), Leu content (I), Phe content (J), Pro content (K) and Met content (L) in 1-MCP treated chives and untreated chives during storage at 20 ^o^C and 3 ^o^C. The content of free amino acids in 1-MCP treated chives stored at 3 ^o^C were not determined. Data are expressed as the mean of triplicate samples. Vertical bars represent the standard errors of the means. The lower and upper case letters indicate difference among the storage time and the treatments of the chive samples respectively.
