# Supplementary material for: 1-Methylcyclopropene Preserves the Quality of Chive (Allium schoenoprasum L.) by Enhancing Its Antioxidant Capacities and Organosulfur Profile during Storage
Source: Foods. 2021 Aug 2;10(8):1792. doi: 10.3390/foods10081792 (PMC8393522; doi:10.3390/foods10081792)
Supplement: Supplementary file 1 [file foods-10-01792-s001.zip › foods-1277033-supplementary.pdf]

SUPPLEMENTARY INFORMATION

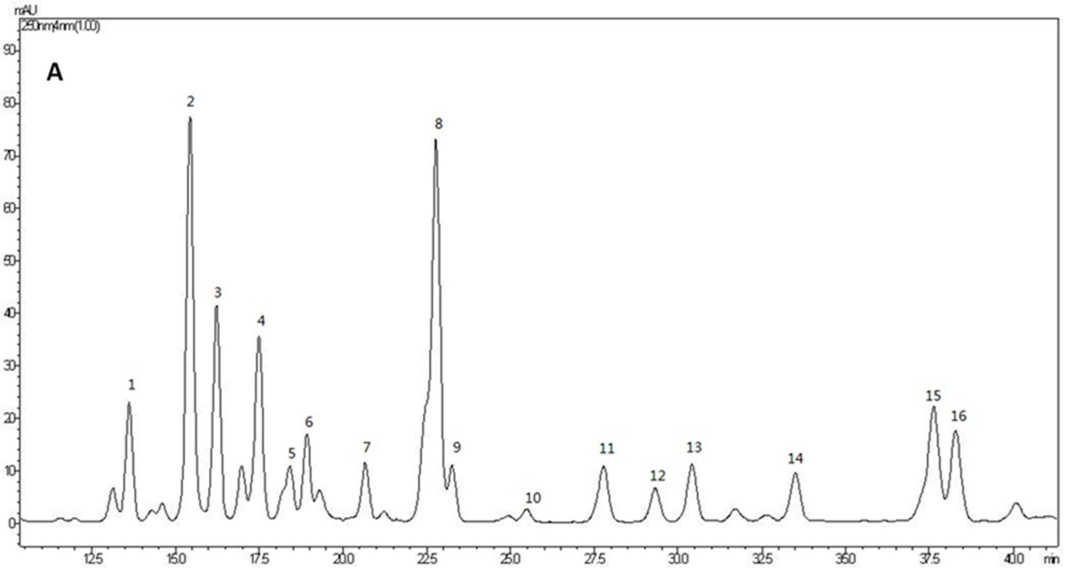

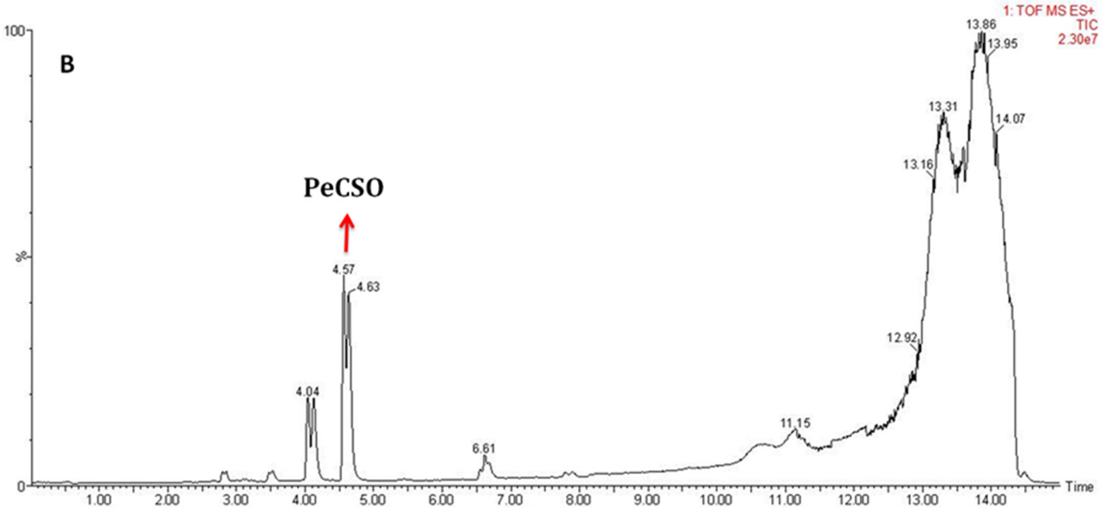

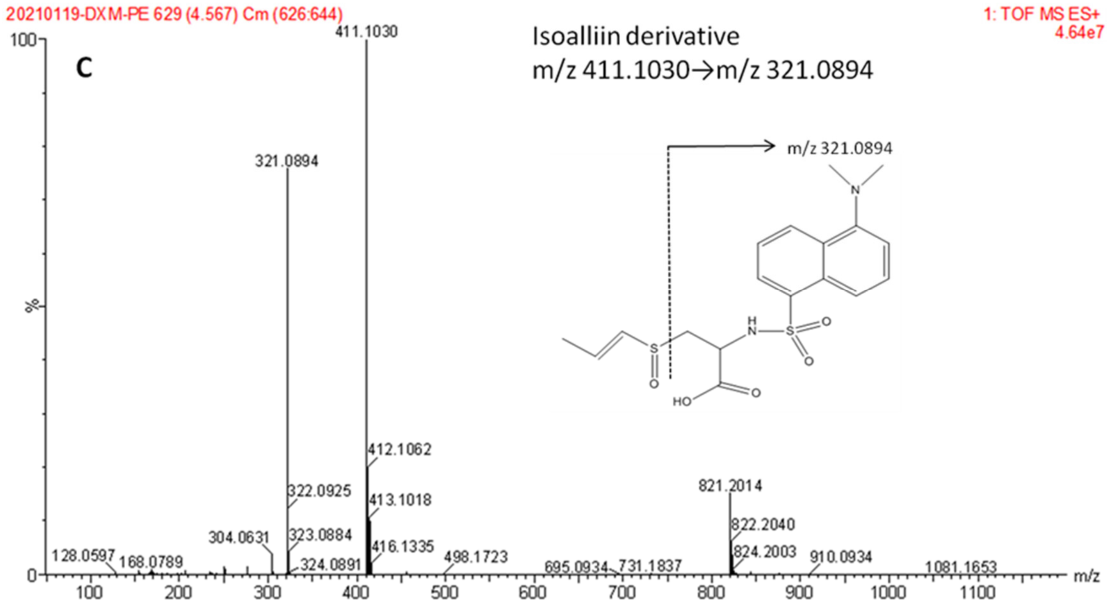

Figure S1. Separation and identification of organosulfur compounds and amino acids. HPLC chromatograms of organosulfur compounds and amino acids in chives (**A**), the peaks as the following: 1.Asn, 2. Gln, 3. Ser, 4. MCSO, 5. Arg, 6. Thr, 7. Ala, 8. PeCSO, 9. ACSO, 10. PCSO, 11. Pro, 12. Met, 13. Val, 14. Trp, 15. Leu, 16. Phe. TIC (**B**) and Mass spectrum (**C**) ($[M+H]^+$) of LC-MS chromatograms of separated peak 8 (a derivative of isoalliin with Dns-Cl) of chives extracts.

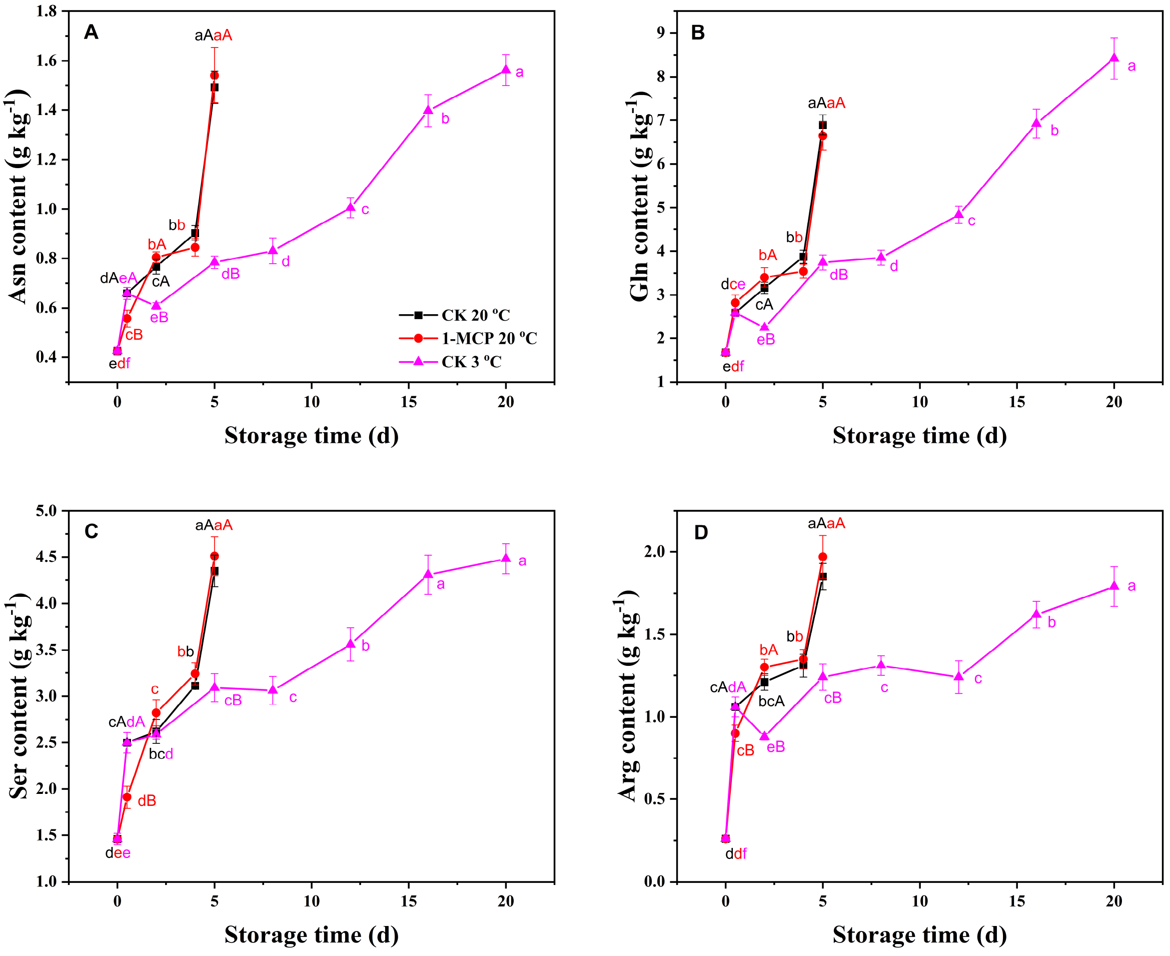

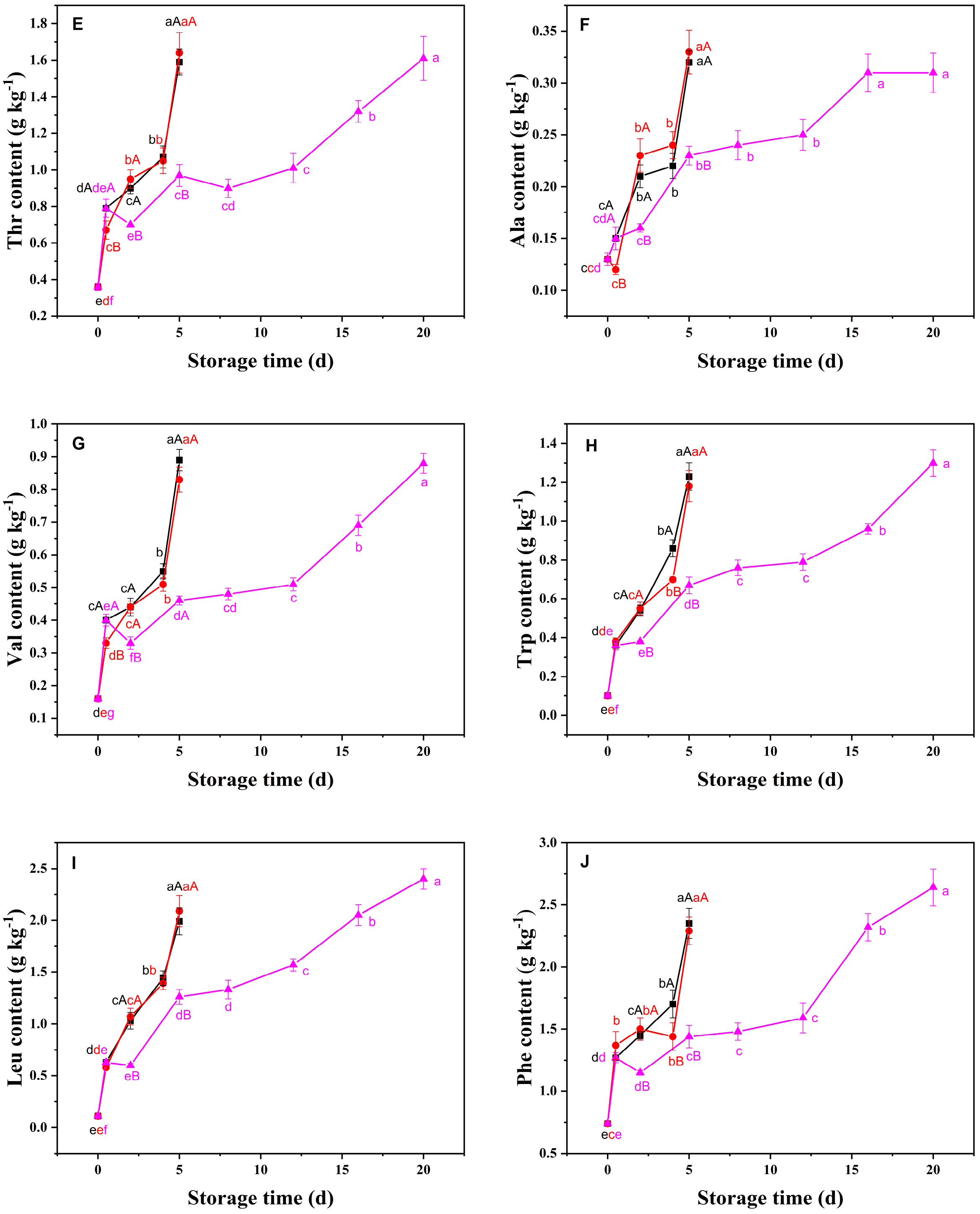
E
Thr content (g kg^{-1})
Storage time (d)
F
Ala content (g kg^{-1})
Storage time (d)
G
Val content (g kg^{-1})
Storage time (d)
H
Trp content (g kg^{-1})
Storage time (d)
I
Leu content (g kg^{-1})
Storage time (d)
J
Phe content (g kg^{-1})
Storage time (d)
0
5
10
15
20

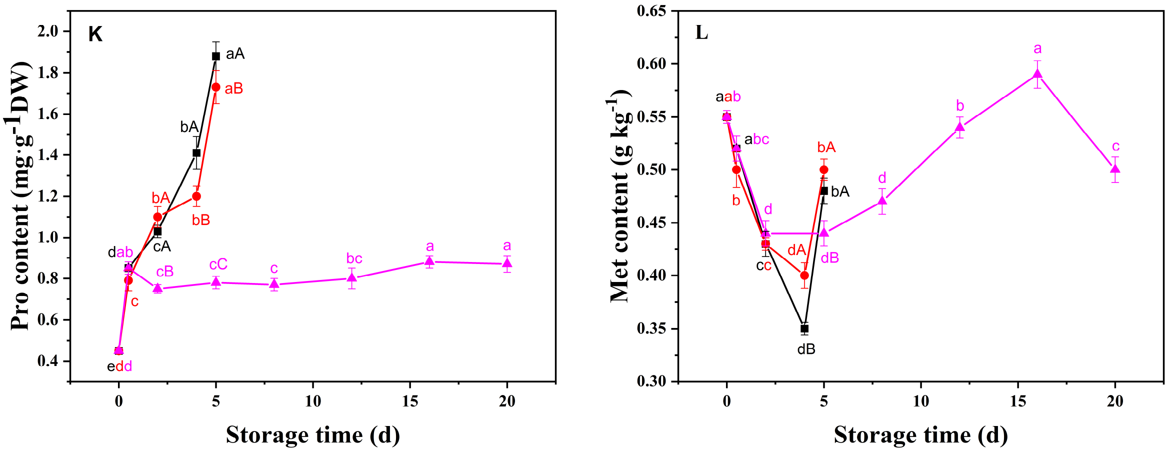

Figure S2. Asn content (**A**), Gln content (**B**), Ser content (**C**), Arg content (**D**), Thr content (**E**), Ala content (**F**), Val content (**G**), Trp content (**H**), Leu content (**I**), Phe content (**J**), Pro content (**K**) and Met content (**L**) in 1-MCP treated chives and untreated chives during storage at 20 °C and 3 °C. The content of free amino acids in 1-MCP treated chives stored at 3 °C were not determined. Data are expressed as the mean of triplicate samples. Vertical bars represent the standard errors of the means. The lower and upper case letters indicate difference among the storage time and the treatments of the chive samples respectively.
